# Profiling of the perturbed metabolomic state of mouse spleen during acute and chronic toxoplasmosis

**DOI:** 10.1186/s13071-017-2282-6

**Published:** 2017-07-18

**Authors:** Xiao-Qing Chen, Chun-Xue Zhou, Hany M. Elsheikha, Shuai He, Gui-Xue Hu, Xing-Quan Zhu

**Affiliations:** 1 Department of Veterinary Medicine, College of Animal Science and Technology, Jilin Agricultural University, Changchun, Jilin Province 130118 People’s Republic of China; 2 State Key Laboratory of Veterinary Etiological Biology, Key Laboratory of Veterinary Parasitology of Gansu Province, Lanzhou Veterinary Research Institute, Chinese Academy of Agricultural Sciences, Lanzhou, Gansu Province 730046 People’s Republic of China; 3 Department of Parasitology, Shandong University School of Basic Medicine, Jinan, Shandong Province 250012 People’s Republic of China; 4 Faculty of Medicine and Health Sciences, School of Veterinary Medicine and Science, University of Nottingham, Sutton Bonington Campus, Loughborough, LE12 5RD UK; 5 College of Animal Science and Technology, Anhui Agricultural University, Hefei, Anhui Province 230036 People’s Republic of China

**Keywords:** *Toxoplasma gondii*, Spleen, Mass spectrometry, Metabolome, Non-targeted metabolomics, Pathway enrichment analysis

## Abstract

**Background:**

*Toxoplasma gondii*, a common opportunistic protozoan, is a leading cause of illness and mortality among immunosuppressed individuals and during congenital infections. Current therapeutic strategies for toxoplasmosis are not fully effective at curtailing disease progression in these cases. Given the parasite ability to influence host immunity and metabolism, understanding of the metabolic alterations in the host’s immune organs during *T. gondii* infection may enhance the understanding of the molecular mechanisms that define the pathophysiology of *T. gondii* infection.

**Methods:**

We investigated the global metabolic changes in the spleen of BALB/c mice at early and late stage of infection with *T. gondii* using LC-MS/MS-based metabolomics. Multivariate data analysis methods, principal components analysis (PCA) and partial least squares discriminant analysis (PLS-DA), were used to identify metabolites that are influenced by *T. gondii* infection.

**Results:**

Multivariate analyses clearly separated the metabolites of spleen of infected and control mice. A total of 132 differential metabolites were identified, 23 metabolites from acutely infected *versus *control mice and 109 metabolites from chronically infected *versus* control mice. Lipids, hormones, lactones, acids, peptides, antibiotics, alkaloids and natural toxins were the most influenced chemical groups. There were 12 shared differential metabolites between acutely infected *versus* control mice and chronically infected *versus* control mice, of which 4,4-Dimethyl-5alpha-cholesta-8,14,24-trien-3beta-ol was significantly upregulated and ubiquinone-8 was significantly downregulated. Major perturbed metabolic pathways included primary bile acid biosynthesis, steroid hormone biosynthesis, biotin metabolism, and steroid biosynthesis, with arachidonic acid metabolism being the most significantly impacted pathway. These metabolic changes suggest a multifactorial nature of the immunometabolic responses of mouse spleen to *T. gondii* infection.

**Conclusions:**

This study demonstrated that *T. gondii* infection can cause significant metabolomic alterations in the spleen of infected mice. These findings provide new insights into the molecular mechanisms that underpin the pathogenesis of *T. gondii* infection.

**Electronic supplementary material:**

The online version of this article (doi:10.1186/s13071-017-2282-6) contains supplementary material, which is available to authorized users.

## Background

Toxoplasmosis is a common opportunistic infection caused by *Toxoplasma gondii*, which can infect almost all warm-blooded vertebrate animals [1]. This parasite is also highly zoonotic, with roughly one-third of the world population reported to be infected [2]. *Toxoplasma gondii* infections of healthy, immunocompetent individuals are usually asymptomatic [3, 4]. However, *T. gondii* can cause significant morbidity and mortality under certain conditions, such as AIDS, bone marrow or solid organ transplantations, or if infection occurs during pregnancy [3, 4]. *Toxoplasma gondii* infection can manifest as an acute infection attributed to the replicating tachyzoite stage, which results in significant immune activation and systemic dissemination to various host tissues [5]. In the presence of an effective immune response, tachyzoites transform into slowly replicating bradyzoites, which exist in the brain and muscle tissue in the form of cysts and this marks the chronic phase of the infection [6, 7]. Control of *T. gondii* replication and transition to the latent form depends on cell-mediated immunity; however, humoral immunity is also critical for resistance to *T. gondii* infection [8, 9].

In recent years, the integration between what used to be traditionally distinct fields, immunology and metabolism, has spurred the emergence of a new discipline called immune system metabolism or “immunometabolism”. Previous studies have shown that metabolic changes in the body are connected to immune regulation. For example, amino acids can modulate the immune function by regulating lymphocyte proliferation and cytokine production [10]. Also, abnormal metabolism of phospholipids, urea, and amino acids can trigger the immune responses and inflammatory processes in Alzheimer’s disease [11]. Interestingly, the interaction between immunity and metabolism has also been shown to play a key role in the pathogenesis of *T. gondii*. Infection with this parasite can evoke major metabolic reprogramming of host cells [12, 13]. In the interim, immunoinflammation is a common feature associated with *T. gondii* infection, in the brain and peripheral circulation [14]. Also, *T. gondii* employs various immunoregulatory mechanisms of evading or subverting host defenses in order to persist as a dormant stage and to ensure transmission to the subsequent host [15]. These mechanisms include, for example, alteration in the architecture of spleen, dysregulation of the expression of cytokines, and generation of anti-parasitic humoral immune response [16].

Surprisingly, investigation of peripheral immune organs that could be affected by systemic perturbations occurring during toxoplasmosis has not been addressed yet. Spleen is a major immune organ playing a critical role in the innate and adaptive immune responses. It is mainly made up of red and white pulp. The white pulp includes B cell and T cell zones, and activates specific immune responses that protect individuals against microbial infection. Knowledge of the metabolic composition of spleen during *T. gondii* infection may reveal the underlying molecular and immune-regulatory processes that operate to counter *T. gondii* infection. Metabolomics has been a powerful tool for the simultaneous quantitative measurement of many metabolites in response of individuals or organisms to drug treatment or other interventions [17] or to study host-parasite interaction [18]. Liquid chromatography coupled to mass spectrometry (LC-MS), including targeted LC-MS and non-targeted LC-MS, has been widely applied to identify and quantify metabolites [12, 13]. We previously employed non-targeted metabolomics to assess the metabolic changes in the brain and serum of mice at different stages of *T. gondii* infection [12, 13]. These studies revealed a profound impact of *T. gondii* on host metabolism, with numerous pathways being affected, and some of the most impacted pathways were related to the metabolism of amino acids, energy metabolism and immune signaling [13].

Given the intricate relationship between immunity and metabolism, and given the importance of spleen in generating anti-*T. gondii* immune response, metabolic fingerprinting of spleen tissues might provide important information about the metabolic alterations that underpin the immunologic responses during *T. gondii* infection. In this work, we hypothesized that spleen exhibits distinct metabolomic patterns in response to *T. gondii* infection, which could result in different spleen functions, particularly affecting the immune response to infection. To test this hypothesis, we investigated, for the first time, changes in the metabolism of spleen of mice infected with *T. gondii* during acute and chronic phases of infection using non-targeted LC-MS/MS metabolomics. Our findings confirmed that using LC-MS/MS-based metabolomics coupled to chemometric methods can provide a powerful approach for discerning metabolic changes in the spleen of mice infected with *T. gondii* and for elucidating infection stage-specific metabolic profiles.

## Methods

### Animal infection

Six-week-old, female, BALB/c mice were obtained from the Laboratory Animal Center of Lanzhou Veterinary Research Institute, Chinese Academy of Agricultural Sciences. Mice were housed in microisolator cages under specific-pathogen free (SPF) conditions, with controlled temperature (22 ± 2 °C), 12 h light/ dark cycles and were given water and standard food pellets *ad libitum*. Mice (*n* = 39) were randomly allocated into two groups: infected group (*n* = 26) and control group (*n* = 13). *Toxoplasma gondii* type II Prugniuad (Pru) strain was maintained in mice *via* oral inoculation of the parasite cysts that were isolated from mice brain tissues 40 days post-infection (dpi). Each mouse in the infected group was infected orally with 10 cysts of *T. gondii* Pru strain in 100 μl phosphate buffered saline solution (PBS). Control mice were gavaged with an equivalent volume of PBS only. All mice were observed daily throughout the entire experiment.

### Tissue collection and detection of infection

To correlate changes in spleen metabolism by stage of infection, we collected spleen samples at 11 and 30 dpi, which were correlated with the acute and chronic stages of infection, respectively. In our previous studies, mice infected with *T. gondii* exhibited acute clinical signs at 7 dpi, which declined gradually until disappearing after 21 dpi [12, 13]. Also, transcriptional changes observed in *T. gondii*-infected mice at 10 and 28 dpi were correlated with acute and chronic infection, respectively [19]. Spleens from uninfected (control) mice were also collected at 30 dpi. Mice were anesthetized by isoflurane inhalation and sacrificed by exsanguination *via* cardiac puncture. Spleen was rapidly removed, rinsed with saline solution (0.9% NaCl *w*/*v*), snap-frozen in liquid nitrogen, and stored at -80 °C until analysis. Tissues from other organs, including brain, blood, liver, lung, small intestine and kidney, were examined for the presence of *T. gondii*, as previously described [20]. Briefly, genomic DNA was extracted from these various tissues using QIAamp® DNA Mini kit following the manufacturer’s instructions (QIAGEN, Hilden, Germany). DNA was then used as a template for PCR to amplify *T. gondii* B1 gene using the specific primers (5′-AAC GGG CGA GTA GCA CCT GAG GAG-3′ and 5′-TGG GTC TAC GTC GAT GGC ATG ACA AC-3′). Positive control (DNA from *T. gondii*) and negative control (deionized water) samples were included in each PCR run. Sections from spleens of 9 mice (3 from each of the acutely infected, chronically infected, and control groups) were collected and processed for routine histopathological examination. Briefly, tissues were fixed in 10% neutral formaldehyde solution for 1 week, dehydrated in a graded series of ethanol, embedded in paraffin wax, cut into 5 μm-thick serial sections on a microtome, stained with hematoxylin-eosin (H&E), and finally examined under an optical microscope (Olympus, Tokyo, Japan).

### Metabolite extraction

A total of 30 spleen samples were collected from two different infection groups (acutely infected, *n* = 10; chronically infected, *n* = 10) plus a control group (*n* = 10). Samples were gradually transferred from -80 °C to -20 °C and then to ﻿4 °C. The organic protein precipitation method was used to extract metabolites. Briefly, 25 mg splenic tissues that were collected from a homogenous mixture of the whole spleen were ground in a mortar in liquid nitrogen. Then, 800 μl methanol/water (1:1) solution and 3 mm (mean diameter) steel beads were added to each sample. Using a TissueLyser bead-mill homogenizer (Qiagen, Hilden, Germany), samples were homogenized *via* vibrating at 60 Hz for 5 min. Subsequently, samples were centrifuged at 25,000× *g* for 10 min at 4 °C. The supernatant of each sample was separated and freeze-dried. Also, 200 μl supernatant of each sample were pooled and used as quality control (QC) samples. Finally, freeze-dried samples were reconstituted, and subjected to mass spectrometry analysis.

### LC-MS/MS analysis

Unbiased metabolomics analysis was performed using an ultra-performance liquid chromatography (UPLC) system (Waters, Milford, USA). The chromatographic separation was carried out using an ACQUITY UPLC BEH C18 column (100 mm × 2.1 mm, 1.7 μm, Waters) with a column temperature of 50 °C and a flow rate of 0.4 ml/min, where the mobile phase contained solvent A (water +0.1% formic acid) and solvent B (acetonitrile +0.1% formic acid). Metabolites were eluted using the following gradient elution conditions: 100% phase A for 0–2 min; 0–100% phase B for ~11 min; 100% phase B for 11–13 min; 100% Phase A for 13–15 min. The loading volume of each sample was 10 μl. The metabolites eluted from the column were detected by a high-resolution tandem mass spectrometer SYNAPT G2 XS QTOF (Waters) in positive and negative ion modes. For positive ion mode, the capillary voltage and the cone voltage were set at 2 kV and 40 V, respectively. For negative ion mode, they were 1 kV and 40 V, respectively. Centroid MSE mode was used to collect the mass spectrometry data. The primary scan ranged from 50 to 1200 Da and the scanning time was 0.2 s. All the parent ions were fragmented using 20–40 eV. The information of all fragments were collected and the time was 0.2 s. In the data acquisition process, the LE signal was gained every 3 s for real-time quality correction. Furthermore, quality control samples (10 samples) were collected to evaluate the stability of the instrument during measurements.

### Multivariate statistical analysis

The raw mass spectrometry data were processed, extracted and the peaks were identified. This procedure involved chromatogram alignment, peak picking, peak area extraction and normalization by commercial software progenesis QI (version 2.2) implementation. Multivariate statistical analyses (principal components analysis, PCA; partial least squares-discriminant analysis, PLS-DA) were performed to discriminate infected mice from control mice. PLS-DA has been widely used in high-dimensional data analysis, especially in the field of metabolomics to maximize group separation [21, 22]. Differentially expressed metabolites were identified on the basis of variable importance in the projection (VIP) threshold ≥ 1 from the PLS-DA model and *P*-values obtained from a two-tailed, Student’s t-test on the normalized peak areas < 0.05. The values of *R* ^2^ and *Q* ^2^ parameters were used to verify the fitness and predictive ability of the model. Fold change (FC) was set to be ≥ 1.2 or ≤ 0.8333. Log_2_ FC based on metabolite abundance was used to assess the levels of variation of the differential metabolites between various mouse groups. Data converted to log_2_ format were used in cluster analysis and generation of heat-maps to show the distinction in the metabolic state between infected mice and controls, and between infected mice at different stages of infection.

### Metabolite identification and pathway analysis

We used the online HMDB (http://www.hmdb.ca/) and KEGG (www.genome.jp/kegg/) databases to identify the metabolites by matching the exact molecular mass data (*m*/*z*) of samples with those from the database. If a mass difference between the observed value and the database value was less than 10 ppm, the metabolite would be identified and the molecular formula of the metabolites would further be validated by the isotopic distribution measurement. Reference standards were used to validate and confirm those significantly changed metabolites by comparing their MS/MS spectra and retention times. Metabolic enrichment analysis was performed to identify and visualize the affected pathway in *T. gondii*-infected mice using the MetPA web tool (http://www.metaboanalyst.ca/) [23].

## Results

### *Toxoplasma gondii* infection in mice

**Fig. 1:**
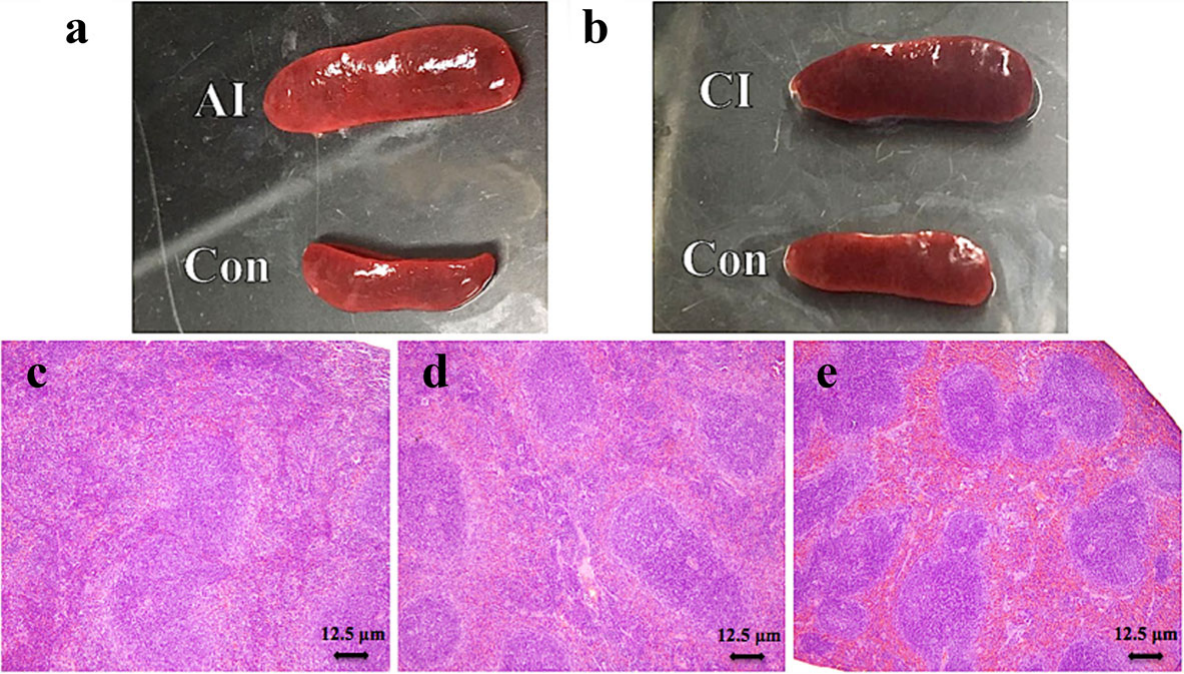
Gross and histopathological characteristics of spleen of mice infected with *Toxoplasma gondii*. **a** The spleen of mice during acute infection (AI) was enlarged compared to normal spleen size of mice in the control group (Con). **b** Mouse spleen during chronic infection (CI) showing slight splenomegaly compared to control (Con) mice. **c** Histopathology of spleen from acutely infected mouse showing a reduction in the white pulp with an expansion of the red pulp compartment. **d** Spleen of a chronically infected mouse showing regression of white pulp and red pulp. **e** Spleen of a healthy mouse showing no histopathological abnormalities. Scale-bars: **c**-**e**, 12.5 μm

Infected mice exhibited mild clinical signs, such as loss of appetite and ruffled hair coat at 7 dpi. These signs progressed and became more evident at 11 dpi, correlating with the acute stage of infection. However, mice began to recover after ~14 dpi and by 30 dpi all mice had developed chronic infection. Therefore, 11 dpi and 30 dpi were selected to represent the acute and chronic phases of infection, respectively. Infection was confirmed in all infected mice by positive PCR results. By contrast, mice in the control group appeared normal and yielded negative PCR results. Histopathology showed splenomegaly in the acutely infected mice (Fig. 1a), but slightly less enlargement in the spleen of chronically infected mice (Fig. 1b). A reduction in the white pulp, but an expansion of the red pulp was observed in the spleen of acutely infected mice (Fig. 1c). By contrast, white pulp hyperplasia with nonreactive red pulp was less prominent in the spleen during chronic infection (Fig. 1d). Spleens of control, uninfected, mice did not show any gross anatomical or histopathological abnormalities (Fig. 1a, b, e).

### Metabolic profiles of mouse groups

**Fig. 2:**
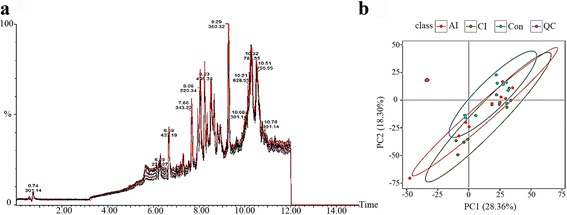
**a** The total ion current (TIC) chromatograms of spleen samples in the positive ion mode (ESI+). **b** PCA scores plot of mice spleens, including acutely infected (AI), chronically infected (CI) and uninfected control (Con) compared to quality control (QC) samples in the positive ion mode (ESI+). Clear separation was detected among the different mice groups and in relation to QC samples

**Fig. 3:**
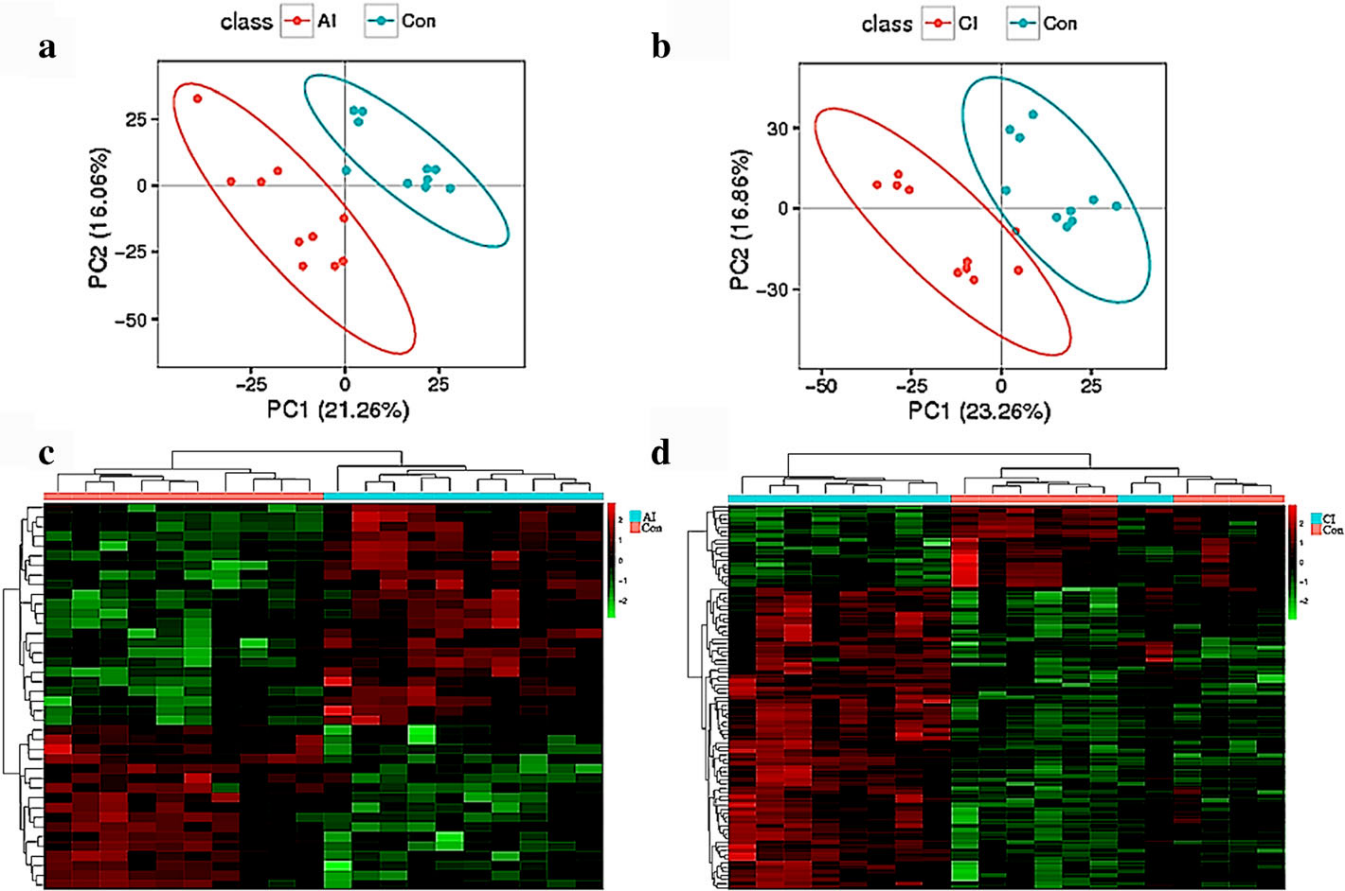
**a, b** Two dimensional PLS-DA score plots of the **a** acutely infected mice and **b** chronically infected mice *vs* control mice in the positive ion mode (ESI+). Each dot represents one spleen sample, projected onto first (horizontal axis) and second (vertical axis) PLS-DA variables. Mice groups are shown in different colors. The ellipse determines the 95% confidence interval. **c**, **d** Heatmaps of the differential metabolites of acutely infected mice **c** and chronically infected mice **d** *vs* control mice in the positive ion mode (ESI+). Red and green indicate values above and below the mean, respectively; black indicates values close to the mean

Mass spectrometry data were analyzed by multivariate statistics to discriminate infected mice from controls. There were 5308 and 6641 molecular features; relative standard deviation (RSD) ≤ 30% ion number of 3702 and 4408; and ratio of 78.30 and 75.08% detected in the positive electrospray ionization (ESI+) mode and negative (ESI-) mode, respectively. Next, we analyzed RSD ≤ 30% ion chromatograms. The total ion chromatograms (TIC) overlap and the PCA scores plot representation of QC samples were performed and confirmed the repeatability and stability of the mass spectrometry measurement, as shown in Fig. 2 (ESI+) and Additional file 1: Figure S1 (ESI-). However, the PCA scores plot did not show any clear separation between the infected and control mice. To better elucidate the metabolic differences, PLS-DA analysis was performed and the score plots are displayed in Fig. 3a, b (ESI+) and Additional file 2: Figure S2 a, b (ESI-). The PLS-DA model of acutely infected *vs* control (*R* ^2^ = 0.913, *Q* ^2^ = 0.5136, ESI+; *R* ^2^ = 0.9237, *Q* ^2^ = 0.4026, ESI-) and chronically infected *vs* control (*R* ^2^ = 0.867, *Q* ^2^ = 0.5184, ESI+; *R* ^2^ = 0.8848, *Q* ^2^ = 0.3388, ESI-) showed a high degree of segregation between the mouse groups. The parameters *R* ^2^ and *Q* ^2^ confirmed the validity of the model. Also, the score plots showed clear discrimination between different infected mouse groups and the control group in both the positive and negative electrospray ionization modes.

### Differential abundance of metabolites by infection phases

**Fig. 4:**
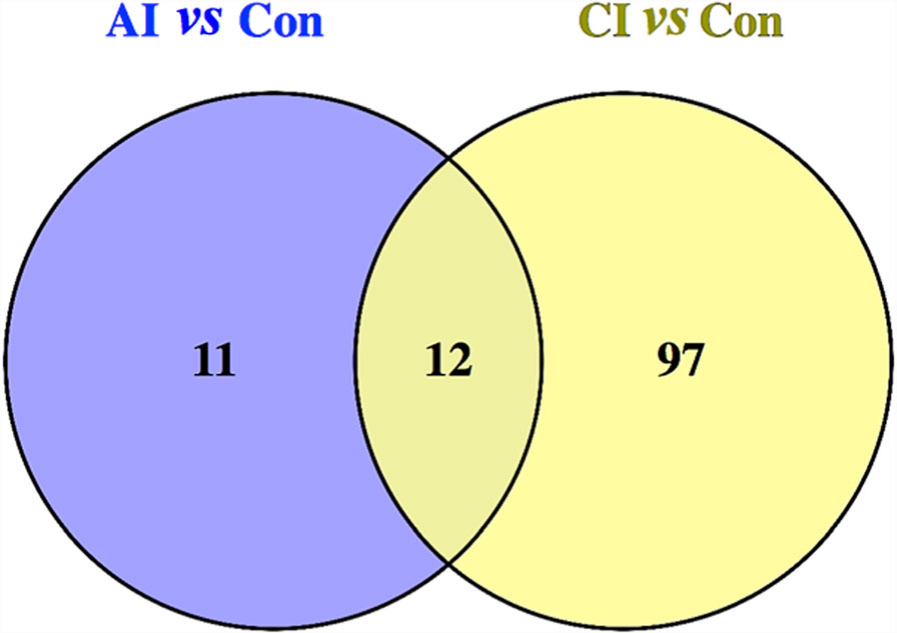
A two-way Venn diagram showing the common and unique metabolites between acutely and chronically infected mice groups *vs* control mice. In total, we found 23 metabolites in acute infection *vs* control (blue), of which 12 metabolites could also be identified in chronically infected mice. Also, we detected 109 metabolites in the chronically infected mice *vs *control (yellow), 12 of which were shared between the groups

The differential metabolites between different mouse groups were categorized according to the stage of infection. The results (Additional file 3: Table S1) show the upregulated and downregulated metabolites between different mouse groups. These differential metabolites between different mouse groups were used for clustering analysis and heatmaps, as shown in Fig. 3c, d (ESI+) and Additional file 2: Figure S2c, d (ESI-). The heatmaps showed a clear difference between acutely infected mice and control mice. Although there was an outlier data point in acutely infected mice in the negative ion mode (Additional file 2: Figure S2a), the same data point clustered with the other points in the same mouse group in the positive ion mode (Fig. 3a). By contrast, chronically infected group was not clearly separated from the control group. In the positive and negative modes, two individuals in the chronically infected group clustered with the control group. Finally, 132 significant metabolites were identified, 23 from acutely infected group *vs * control and 109 from chronically infected *vs* control, as shown in Additional file 4: Table S2 and Additional file 5: Table S3, respectively. These metabolites included lipids, hormones, lactones, acids, peptides, antibiotics, alkaloids and natural toxins. There were 32 metabolites involved in wide range metabolic pathways (Table 1). Venn diagram was developed to correlate metabolites to the infection phase (Fig. 4). The acutely infected group *vs* control and chronically infected *vs* control shared 12 differential metabolites. Also, 4,4-Dimethyl-5alpha-cholesta-8,14,24-trien-3beta-ol was significantly upregulated and Ubiquinone-8 was significantly downregulated. However, levels of arachidonic acid (AA) were different between acute and chronic infection phases (Table 2).

**Table 1:** List of identified differential metabolites involved in the perturbed metabolic pathways during acute and chronic phases of *Toxoplasma gondii* infection

Mice group	Ionization method	m/z - RT	Metabolites	VIP	FC	*q*-value	Metabolic pathways
AI *vs* Con	ESI (+)	369.205–5.980	Corticosterone	2.843	3.049	0.017	Steroid hormone biosynthesis; Regulation of lipolysis in adipocytes; Aldosterone synthesis and secretion
		433.342–7.128	4,4-Dimethyl-5alpha-cholesta-8,14,24-trien-3beta-ol	5.216	9.497	0.040	Steroid biosynthesis
		299.197–7.263	Stearidonic acid	1.819	0.732	0.025	alpha-Linolenic acid metabolism
		453.293–7.612	7alpha-Hydroxy-3-oxo-4-cholestenoate	1.462	0.704	0.026	Primary bile acid biosynthesis
		454.390–7.684	3alpha,7alpha,12alpha,26-Tetrahydroxy-5beta-cholestane	1.369	0.331	0.011	Primary bile acid biosynthesis
		331.224–7.727	17alpha,21-Dihydroxypregnenolone	1.907	0.550	0.035	Steroid hormone biosynthesis
		709.559–8.548	Ubiquinone-8	4.574	0.0565	0.040	Ubiquinone and other terpenoid-quinone biosynthesis
	ESI (−)	303.232–7.199	Arachidonic acid	1.271	0.652	0.045	Arachidonic acid metabolism; Linoleic acid metabolism; Biosynthesis of unsaturated fatty acids; Vascular smooth muscle contraction; Platelet activation; Fc epsilon RI signaling pathway; Fc gamma R-mediated phagocytosis; Retrograde endocannabinoid signaling; Serotonergic synapse; Long-term depression; Inflammatory mediator regulation of TRP channels; GnRH signaling pathway; Ovarian steroidogenesis; Oxytocin signaling pathway; Regulation of lipolysis in adipocytes; Aldosterone synthesis and secretion
		453.161–8.588	Methotrexate	1.537	0.731	0.047	Bile secretion
CI *vs* Con	ESI (+)	433.342–7.128	4,4-Dimethyl-5alpha-cholesta-8,14,24-trien-3beta-ol;	4.660	6.459	0.046	Steroid biosynthesis
		454.390–7.684	3alpha,7alpha,12alpha,26-Tetrahydroxy-5beta-cholestane	1.185	0.385	0.014	Primary bile acid biosynthesis
		385.344–7.886	Cerebrosterol	2.976	8.236	0.046	Primary bile acid biosynthesis
		361.150–8.322	Neamine	3.163	4.633	0.023	Butirosin and neomycin biosynthesis; Biosynthesis of antibiotics
		305.214–8.519	19-Hydroxytestosterone	1.896	1.669	0.049	Steroid hormone biosynthesis
		351.229–8.533	Docosahexaenoic acid(DHA)	1.113	1.331	0.033	Biosynthesis of unsaturated fatty acids
		709.559–8.548	Ubiquinone-8	7.038	0.019	0.002	Ubiquinone and other terpenoid-quinone biosynthesis
		391.284–8.943	Bile acid	1.342	1.470	0.037	Fat digestion and absorption; Vitamin digestion and absorption
		365.194–8.994	Phorbol	1.464	1.483	0.029	Inflammatory mediator regulation of TRP channels
		419.350–9.386	7alpha,27-Dihydroxycholesterol	1.509	2.112	0.032	Primary bile acid biosynthesis
		425.339–9.728	7alpha-Hydroxycholesterol	2.029	2.228	0.006	Primary bile acid biosynthesis
		343.195–8.563	Arachidonic acid	2.132	1.757	0.015	Arachidonic acid metabolism; Linoleic acid metabolism; Biosynthesis of unsaturated fatty acids; Vascular smooth muscle contraction; Platelet activation; Fc epsilon RI signaling pathway; Fc gamma R-mediated phagocytosis; Retrograde endocannabinoid signaling; Serotonergic synapse; Long-term depression; Inflammatory mediator regulation of TRP channels; GnRH signaling pathway; Ovarian steroidogenesis; Oxytocin signaling pathway; Regulation of lipolysis in adipocytes; Aldosterone synthesis and secretion;
	ESI (−)	295.227–10.292	(9S)-Hydroxyoctadecadienoic acid	1.707	0.702	0.020	Linoleic acid metabolism; PPAR signaling pathway
		317.210–7.655	Leukotriene A4	1.364	1.623	0.020	Arachidonic acid metabolism; Serotonergic synapse
		315.195–8.218	15-Deoxy-delta-12,14-PGJ2	1.834	1.942	0.005	Arachidonic acid metabolism
		319.189–8.325	Ubiquinol	2.203	2.424	0.038	Oxidative phosphorylation
		301.215–8.353	(5Z,7E,9E,14Z,17Z)-Eicosapentaenoate	3.001	3.042	0.017	Inflammatory mediator regulation of TRP channels
		289.216–8.328	Androstenediol	2.718	3.058	0.048	Steroid hormone biosynthesis; Ovarian steroidogenesis
		550.264–8.439	Taurocholate	1.654	1.936	0.023	Primary bile acid biosynthesis; Taurine and hypotaurine metabolism; Bile secretion
		371.179–8.511	Biocytin	1.024	0.806	0.030	Biotin metabolism; Vitamin digestion and absorption
		543.151–8.525	Premithramycin A1	2.026	0.598	0.030	Biosynthesis of antibiotics
		416.316–8.571	N-Oleoyl dopamine	2.167	3.359	0.024	Neuroactive ligand-receptor interaction
		453.161–8.588	Methotrexate	1.686	0.701	0.008	Bile secretion
		569.166–8.588	5″-Phosphoribostamycin	1.823	0.538	0.045	Butirosin and neomycin biosynthesis
		305.247–8.774	(8Z,11Z,14Z)-Icosatrienoic acid	1.630	1.519	0.031	Linoleic acid metabolism; Biosynthesis of unsaturated fatty acids
		307.262–9.009	Icosadienoic acid	1.767	1.819	0.006	Biosynthesis of unsaturated fatty acids
		337.326–9.024	14,15-DHET	1.702	1.557	0.029	Arachidonic acid metabolism; Serotonergic synapse
		283.262–9.238	Octadecanoic acid	1.134	1.207	0.007	Fatty acid biosynthesis; Biosynthesis of unsaturated fatty acids

*Abbreviations*: m/z - RT, MS and retention time; VIP, variable importance for projection; FC, fold change; *q*-value, adjusted *P*-value calculated by two-tailed Wilcoxon rank-sum tests after false discovery rate correction

**Table 2:** List of common metabolites in acute and chronic phases of *Toxoplasma gondii* infection

KEGG.ID	Metabolite	Differentially expressed metabolite^a^	
		Acutely infected	Chronically infected
C11455	4,4-Dimethyl-5alpha-cholesta-8,14,24-trien-3beta-ol	↑	↑
C05446	3alpha,7alpha,12alpha,26-Tetrahydroxy-5beta-cholestane	↓	↓
C13804	ORG 20599	↓	↓
C17569	Ubiquinone-8	↓	↓
C11606	NAc-FnorLRF-amide	↑	↑
C00219	Arachidonic acid	↓	↑
C16147	Glycosyl-4,4′-diaponeurosporenoate	↑	↑
C16885	Gambieric acid A	↑	↑
C09261	Disenecionyl cis-khellactone	↑	↑
C10458	Furcatin	↑	↑
C01937	Methotrexate	↓	↓
C14668	Cortancyl	↑	↑

^a^↑, upregulated; ↓, downregulated

### Metabolic pathways affected by infection phases

**Fig. 5:**
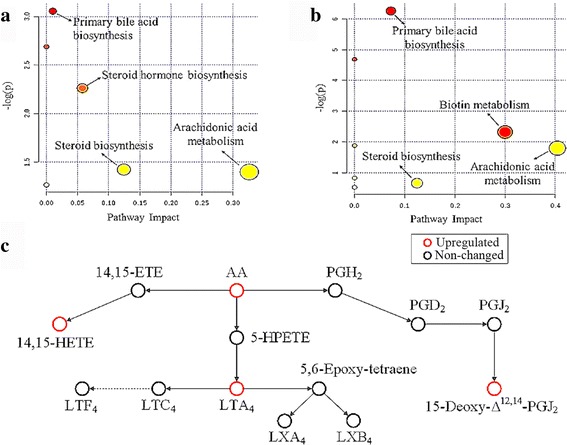
Pathway analysis of the differential metabolites during acute and chronic infection. Metabolite features with putative identification were analyzed using MetaboAnalyst for potential impact on metabolic pathways in the acute phase **a** and chronic phase **b**. Small *P*-value and large pathway impact factor indicate that the pathway is greatly influenced, such as arachidonic acid metabolism pathway, which was highly impacted during acute and chronic infection. **c** A schematic illustration of the arachidonic acid metabolism pathway during chronic infection. Red and black circles represent upregulated and unaltered metabolites, respectively. As shown, arachidonic acid (AA), leukotriene A4 (LTA4), 14,15-HETE, and 15-deoxy-Δ^12,14^-PGJ_2_ were upregulated

Metabolite enrichment analysis was performed and several metabolic pathways were found to be influenced by infection. As shown in Fig. 5a and Table 3, the metabolic pathways identified during acute infection involved primary bile acid biosynthesis, steroid hormone biosynthesis, arachidonic acid metabolism and steroid biosynthesis, among others. Some of the impacted pathways during chronic infection involved primary bile acid biosynthesis, steroid hormone biosynthesis, biotin metabolism, arachidonic acid metabolism and steroid biosynthesis (Fig. 5b, Table 4). The most deeply impacted pathway during both acute and chronic infection was the arachidonic acid metabolism. An illustration of AA metabolism pathway is provided to show specific metabolites dysregulated during chronic infection (Fig. 5c).

**Table 3:** Summary of the pathway analysis using MetaboAnalyst during acute *Toxoplasma gondii* infection

Metabolic pathway	Raw *P*-value^a^	-log(p)	Impact^b^
Arachidonic acid metabolism	0.2473	1.3971	0.3260
Steroid biosynthesis	0.2413	1.4219	0.1245
Steroid hormone biosynthesis	0.1041	2.2623	0.0578
Primary bile acid biosynthesis	0.0471	3.0561	0.0102

^a^Raw *P*-value is the original *P*-value calculated from the enrichment analysis

^b^Impact is the pathway impact value calculated from pathway topology analysis

**Table 4:** Summary of the pathway analysis using MetaboAnalyst during chronic *Toxoplasma gondii* infection

Metabolic pathway	Raw *P*-value^a^	-log(p)	Impact^b^
Arachidonic acid metabolism	0.1664	1.7935	0.4042
Biotin metabolism	0.0984	2.3191	0.3000
Steroid biosynthesis	0.5194	0.6551	0.1245
Primary bile acid biosynthesis	0.0019	6.2581	0.0724

^a^Raw *P*-value is the original *P* value calculated from the enrichment analysis

^b^Impact is the pathway impact value calculated from pathway topology analysis

## Discussion

The aim of this work was to elucidate the effect of *T. gondii* infection on the metabolism of mouse spleen, a key organ in regulating immune response against *T. gondii* infection. LC-MS/MS-based metabolomics and multivariate statistical analyses revealed new metabolic changes in the spleen of *T. gondii*-infected mice compared with uninfected mice and identified metabolic signatures that differentiated between the acute infection and chronic infection.

### Stage-specific metabolic signatures

A total of 389 differential ions were screened based on using VIP scores (VIP > 1) of individual metabolites obtained from the PLS-DA model. We succeeded to identify 259 of the 389 differential ions, which highlights the challenges associated with the identification of metabolites. We used heatmaps to present the results of these differential ions between different mouse groups. The heatmaps showed a clear difference between acutely infected group and the control group (Fig. 3c, Additional file 2: Figure S2c), but chronically infected group was not fully distinct from the control group (Fig. 3d, Additional file 2: Figure S2d). In the positive and negative modes, two mice in the chronically infected group were clustered with mice in the control group, which could be attributed to inter-individual variations among chronically infected mouse group. Also, converging metabolic responses between these two chronically infected and control mice is probably due to homeostatic recovery that might have occurred as infection progressed to the chronic phase. Interestingly, we identified 132 significantly altered metabolites, 23 from acutely infected *vs* control and 109 from chronically infected *vs* control mice (Additional file 4: Table S2, Additional file 5: Table S3), suggesting that as infection progressed the number of differential metabolites increased. This result disagrees with previous metabolomic profiling of serum [12] and brain [13] of *T. gondii*-infected mice where the most predominant metabolic changes, compared with control mice, happened at an early stage of infection. These distinct temporal metabolic patterns between metabolomics studies can be attributed to organ-specific metabolomics organization (i.e. different repertoire of small molecules present in different organs). Also, the metabolomic response to infection of a major peripheral immune organ such as spleen, is expected to be different from that of the serum [12] or brain [13].

There were 12 shared differential metabolites between the acute and chronic infection phases (Table 2, Fig. 4). 4,4-Dimethyl-5alpha-cholesta-8,14,24-trien-3beta-ol was significantly upregulated and ubiquinone-8 was significantly downregulated, suggesting that these two metabolites might play important roles during *T. gondii* infection. The upregulation of 4,4-Dimethyl-5alpha-cholesta-8,14,24-trien-3beta-ol during both acute and chronic phases might be related to the regulatory effect of this molecule on host cell meiosis [24]. Expression patterns of AA were distinct between the acute and chronic infection phases (i.e. infection stage-specific). As shown in Additional file 4: Table S2 and Additional file 5: Table S3, in the two infection phases, most of the identified differential metabolites were lipids, which are essential for the biogenesis of cell and parasite membranes in order to ensure parasite’s survival and replication within host cells [25].

We chose spleen in this study because of its importance in immune surveillance during infection. Spleen comprises two morphologically and functionally distinct regions (red pulp and white pulp) and contains multiple subsets of specialized myeloid and dendritic cells. However, a major drawback of splenic tissue is its complex and dynamic cellular heterogeneity especially in response to infection. In the present study, *T. gondii* infection induced spatial alterations in the composition of splenic cellular populations and splenomegaly was a relatively sub-fatal complication of infection. These changes could have a profound effect on the metabolome of this tissue and consequently make it hard to dissociate the direct effect of infection on metabolite expression patterns from the contribution of the anatomical abnormalities associated with infection. Therefore, it is possible that changes we observed in the levels of metabolites is a consequence of the differential contributions of cellular populations of spleen from acute, chronic and control mice rather than the result of actual changes in the metabolic activity in spleen cells triggered by *T. gondii* infection per se. Precise mechanisms, however, remain unclear and the specific contributions of the metabolites of the red and white pulps to the alterations in the levels of metabolites observed during *T. gondii* infection remains to be clarified.

### The impact on host defense mechanisms

In the acute phase, the level of corticosterone was markedly increased with log_2_ fold change of 3.049. Hormones are very important signaling molecules in mammals and are fundamental for their metabolic and immune homeostasis [26]. Corticosterone is vital in the metabolism of carbohydrates, fatty acids and amino acids. The markedly altered corticosterone might reflect variations in the energy metabolism in the spleen of infected mice, which might be triggered by illness-related anorexia. Ubiquinol of the oxidative phosphorylation pathway was probably upregulated to supply energy needed by the body to balance its metabolic status during the latent phase of infection. The anti-fungal molecule gambieric acids A [27] and the antibiotic molecules, neamine and difloxacin (INN), were also significantly upregulated. Additionally, AA, Phorbol and (5Z,7E,9E,14Z,17Z)-Eicosapentaenoate, which act as inflammatory mediators, were upregulated. It is likely that spleen defends against *T. gondii* infection not only by specific immune processes, but also by eliciting different metabolic reactions as part of the innate immunity to limit the infection.

### *Toxoplasma gondii* infection disturbs eicosanoid metabolism

The altered metabolic pathways identified involved primary bile acid biosynthesis, steroid biosynthesis and arachidonic acid metabolism. Steroid hormones are involved in a variety of physiological processes; relevant to *T. gondii* pathogenesis are the immunoregulatory and anti-inflammatory effects of steroids, which can influence the host immune responses to infection [28]. Our results also revealed AA metabolism, the main precursor of eicosanoid hormones, as the most significantly affected metabolic pathway by *T. gondii* during acute and chronic infection. At 11 dpi, the level of AA decreased, indicating down-regulation of AA metabolism during acute infection. By contrast, at 30 dpi the level of AA was significantly upregulated in the chronic phase (Fig. 5c). The major inflammatory mediators, LTA4, 14,15-HETE, and 15-deoxy-Δ^12,14^-PGJ_2_ [29–31], were also up-regulated. In response to an inflammatory stimulus, AA, the main polyunsaturated fatty acid present in the phospholipid of cell membranes, is released and metabolized to a series of eicosanoids, including the inflammatory leukotrienes and prostanoids (e.g. prostaglandins, prostacyclins and thromboxanes) [32]. AA and its eicosanoid metabolites play an important role in the regulation of many cellular processes, such as cell survival, angiogenesis, chemotaxis, mitogenesis, apoptosis and migration [33, 34]. Elevated level of AA in the supernatant of *T. gondii*-infected cultured J774A.1 cells was assumed to be triggered by increased Phospholipase A production in order to release AA *via* decomposing the host cell membrane phospholipids, thus promoting the parasites invasion by increasing the host cell membrane permeability and fluidity [35]. Likewise, *T. gondii* was shown to increase AA concentration and agglutination of microfilaments in phagocytic host cells to accelerate the parasite’s invasion [35].

## Conclusions

We employed global LC-MS/MS-based metabolomics to detect differential metabolites in the spleen of mice infected with *T. gondii* and to identify changes in metabolic pathways with direct relevance to the parasite pathogenesis. Our research demonstrated that significant metabolomic impairments occur in the spleen of mice infected with *T. gondii*. These included hormones, lactones, acids, peptides, antibiotics, alkaloids, natural toxins and others. Abnormal metabolism of these metabolites could play various roles in immune response and inflammatory reaction during *T. gondii* infection. Our study also revealed infection stage-specific metabolites, such as arachidonic acid. More differentially expressed metabolites were detected in the chronic phase compared with the acute phase. Further, we detected perturbations in biochemical pathways, including primary bile acid biosynthesis, steroid hormone biosynthesis, biotin metabolism, arachidonic acid metabolism and steroid biosynthesis, involved in the proper functioning of the spleen, which might underpin the dysregulation in systemic immunity in toxoplasmosis. The knowledge of spleen metabolomic differences by disease severity (acute *vs* chronic stage of *T. gondii* infection) has potential clinical implications in the development of optimal therapies.

## Additional files

Additional file 1: Figure S1. a The total ion current (TIC) chromatograms of spleen samples in the negative ion mode (ESI-). b PCA scores plot of mouse’s spleen samples, including acutely infected (AI), chronically infected (CI) and uninfected control (Con) compared to quality control (QC) samples in the negative ion mode (ESI-). (TIFF 281 kb)

Additional file 2: Figure S2. a, b PLS-DA score plots of the (a) acutely infected mice and (b) chronically infected mice *vs* control mice in the negative ion mode (ESI-). c, d Heatmaps of the differential metabolites of (c) acutely infected mice and (d) chronically infected mice *vs* control mice in the negative ion mode (ESI-). (TIFF 470 kb)

Additional file 3: Table S1. Summary results of the differential ions. (DOC 32 kb)

Additional file 4: Table S2. List of metabolites identified during the acute phase of *Toxoplasma gondii* infection. (DOC 54 kb)

Additional file 5: Table S3. List of metabolites identified during the chronic phase of *Toxoplasma gondii* infection. (DOCX 33 kb)

## Abbreviations

AA: Arachidonic acid
AI: Acutely infected
CI: Chronically infected
Con: Uninfected control
dpi: Days post-infection
ESI-: Negative electrospray ionization
ESI+: Positive electrospray ionization
FC: Fold change
LTA4: Leukotriene A4
PCA: Principal components analysis
PLS-DA: Partial least squares discriminant analysis
QC: Quality control
TIC: Total ion chromatograms
VIP: Variable importance in the projection
