# Supplementary figures and images for: Profiling of the perturbed metabolomic state of mouse spleen during acute and chronic toxoplasmosis
Source: Parasit Vectors. 2017 Jul 18;10:339. doi: 10.1186/s13071-017-2282-6 (PMC5516376; doi:10.1186/s13071-017-2282-6)

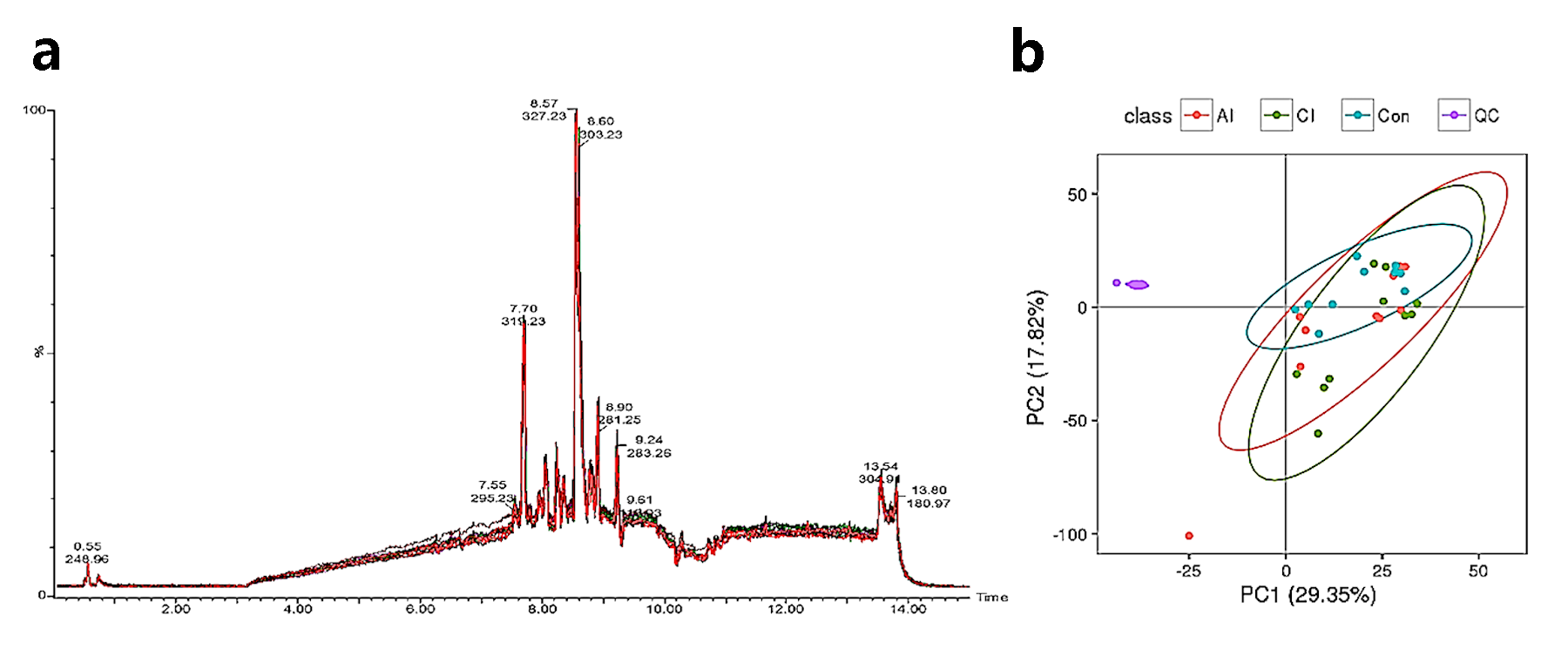

Supplement: Supplementary file 1 — a The total ion current (TIC) chromatograms of spleen samples in the negative ion mode (ESI-). b PCA scores plot of mouse’s spleen samples, including acutely infected (AI), chronically infected (CI) and uninfected control (Con) compared to quality control (QC) samples in the negative ion mode (ESI-). (TIFF 281 kb) [file 13071_2017_2282_MOESM1_ESM.tif]

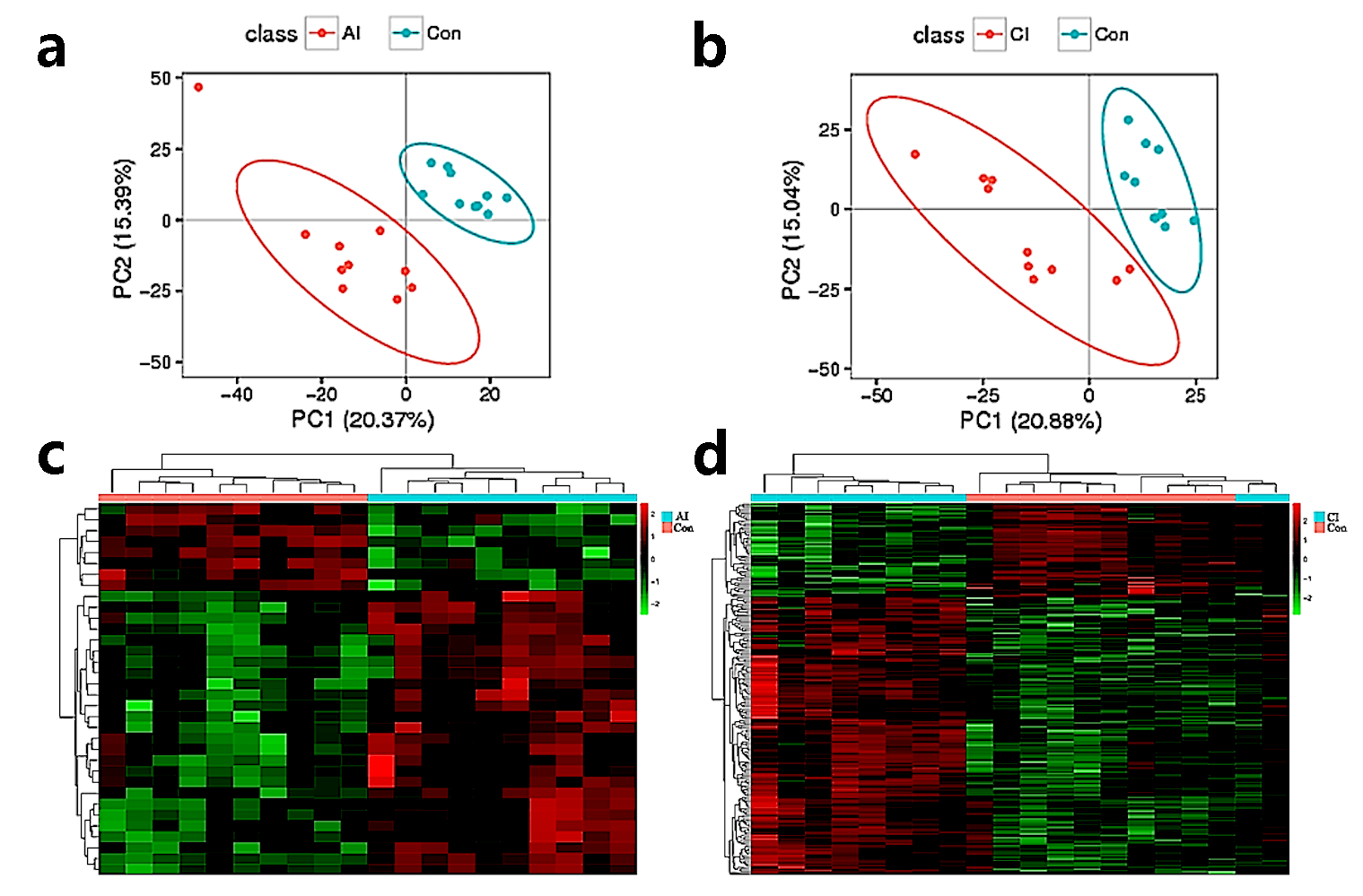

Supplement: Supplementary file 2 — a, b PLS-DA score plots of the (a) acutely infected mice and (b) chronically infected mice vs control mice in the negative ion mode (ESI-). c, d Heatmaps of the differential metabolites of (c) acutely infected mice and (d) chronically infected mice vs control mice in the negative ion mode (ESI-). (TIFF 470 kb) [file 13071_2017_2282_MOESM2_ESM.tif]
