# Supplementary material for: Profiling of the perturbed metabolomic state of mouse spleen during acute and chronic toxoplasmosis
Source: Parasit Vectors. 2017 Jul 18;10:339. doi: 10.1186/s13071-017-2282-6 (PMC5516376; doi:10.1186/s13071-017-2282-6)
Supplement: Supplementary file 3 — Summary results of the differential ions. (DOC 32 kb) [file 13071_2017_2282_MOESM3_ESM.doc]

| Ionization method | Mice  group^*^ | Differential ion number | Identified  metabolites | Up^†^ | Down^†^ |
| --- | --- | --- | --- | --- | --- |
| ESI (+) | AI vs Con | 40 | 14 | 23 | 17 |
| ESI (+) | CI vs Con | 93 | 35 | 73 | 20 |
| ESI (-) | AI vs Con | 34 | 8 | 26 | 8 |
| ESI (-) | CI vs Con | 222 | 75 | 166 | 56 |

**Additional file 3: Table S1** Summary results of the differential ions

**^*^** AI, CI and Con indicate acutely infected, chronically infected and control, respectively.

**^†^**  Up and down refer to up-regulated and down-regulated differential ion number.
