# Supplementary material for: Profiling of the perturbed metabolomic state of mouse spleen during acute and chronic toxoplasmosis
Source: Parasit Vectors. 2017 Jul 18;10:339. doi: 10.1186/s13071-017-2282-6 (PMC5516376; doi:10.1186/s13071-017-2282-6)
Supplement: Supplementary file 4 — List of metabolites identified during the acute phase of Toxoplasma gondii infection. (DOC 54 kb) [file 13071_2017_2282_MOESM4_ESM.doc]

| Ionization method | m/z - RT | KEGG.ID | Metabolites | VIP | F.C. | q-value |
| --- | --- | --- | --- | --- | --- | --- |
| ESI (+) | 212.069-5.438 | C07437 | Phensuximide | 2.070 | 0.423 | 0.011 |
|  | 369.205-5.980 | C02140 | Corticosterone | 2.843 | 3.049 | 0.017 |
|  | 433.342-7.128 | C11455 | 4,4-Dimethyl-5alpha-cholesta-8,14,24-trien-3beta-ol | 5.216 | 9.497 | 0.040 |
|  | 448.343-7.199 | C10811 | Jervine | 1.178 | 0.707 | 0.026 |
|  | 299.197-7.263 | C16300 | Stearidonic acid | 1.819 | 0.732 | 0.025 |
|  | 453.293-7.612 | C17337 | 7alpha-Hydroxy-3-oxo-4-cholestenoate | 1.462 | 0.704 | 0.026 |
|  | 475.269-7.670 | C09086 | Diterpenoid EF-D | 1.148 | 0.718 | 0.018 |
|  | 454.390-7.684 | C05446 | 3alpha,7alpha,12alpha,26-Tetrahydroxy-5beta-cholestane | 1.369 | 0.331 | 0.011 |
|  | 455.306-7.684 | C13804 | ORG 20599 | 1.163 | 0.731 | 0.012 |
|  | 331.224-7.727 | C05487 | 17alpha,21-Dihydroxypregnenolone | 1.907 | 0.550 | 0.035 |
|  | 709.559-8.548 | C17569 | Ubiquinone-8 | 4.574 | 0.0565 | 0.040 |
|  | 623.366-8.592 | C11606 | NAc-FnorLRF-amide | 1.514 | 1.441 | 0.041 |
|  | 655.574-9.204 | C18137 | 2,2',4,4',5,5'-Hexabromodiphenyl ether | 1.088 | 1.284 | 0.048 |
|  | 585.413-9.676 | C12001 | 5-O-beta-D-Mycaminosyltylactone | 3.771 | 0.228 | 0.004 |
|  | 599.430-9.805 | C20785 | Eleutheroside A | 1.782 | 0.696 | 0.023 |
| ESI (-) | 303.232-7.199 | C00219 | Arachidonic acid | 1.271 | 0.652 | 0.045 |
|  | 593.354-8.018 | C16147 | Glycosyl-4,4'-diaponeurosporenoate | 3.931 | 6.124 | 0.009 |
|  | 1091.612-8.032 | C16885 | Gambieric acid A | 3.003 | 3.545 | 0.016 |
|  | 325.215-8.353 | C15190 | 12alpha-Methylpregna-4,9(11)-diene-3,20-dione | 1.928 | 1.811 | 0.043 |
|  | 425.163-8.553 | C09261 | Disenecionyl cis-khellactone | 1.253 | 1.321 | 0.008 |
|  | 427.158-8.553 | C10458 | Furcatin | 1.504 | 1.419 | 0.036 |
|  | 453.161-8.588 | C01937 | Methotrexate | 1.537 | 0.731 | 0.047 |
|  | 399.184-9.224 | C14668 | Cortancyl | 1.383 | 1.364 | 0.016 |

**Additional file 4: Table S2** List of differential metabolites identified during the acute phase of *Toxoplasma gondii* infection

*Abbreviations: m/z – RT, MS and retention time; VIP, variable importance for projection; F.C., Fold change; q-value, adjusted p value calculated by two-tailed Wilcoxon rank-sum tests after false discovery rate correction.*
