# Supplementary material for: Profiling of the perturbed metabolomic state of mouse spleen during acute and chronic toxoplasmosis
Source: Parasit Vectors. 2017 Jul 18;10:339. doi: 10.1186/s13071-017-2282-6 (PMC5516376; doi:10.1186/s13071-017-2282-6)
Supplement: Supplementary file 5 — List of metabolites identified during the chronic phase of Toxoplasma gondii infection. (DOCX 33 kb) [file 13071_2017_2282_MOESM5_ESM.docx]

**Additional file 5: Table S3** List of differential metabolites identified during chronic *Toxoplasma gondii* infection

| Ionization method | m/z - RT | KEGG.ID | metabolites | VIP | F.C. | q-value |
| --- | --- | --- | --- | --- | --- | --- |
| ESI (+) | 641.437-10.844 | C07554 | Pipecuronium | 3.799 | 0.036 | 0.049 |
|  | 617.513-11.090 | C13861 | 1-Hexadecanoyl-2-(9Z-octadecenoyl)-sn-glycerol | 3.568 | 0.103 | 0.045 |
|  | 433.342-7.128 | C11455 | 4,4-Dimethyl-5alpha-cholesta-8,14,24-trien-3beta-ol | 4.660 | 6.459 | 0.046 |
|  | 408.287-7.377 | C15013 | 3beta-(1-Pyrrolidinyl)-5alpha-pregnane-11,20-dione | 1.327 | 0.605 | 0.030 |
|  | 470.385-7.462 | C16890 | 5alpha-Cyprinol | 1.565 | 0.356 | 0.026 |
|  | 454.390-7.684 | C05446 | 3alpha,7alpha,12alpha,26-Tetrahydroxy-5beta-cholestane | 1.185 | 0.385 | 0.014 |
|  | 455.306-7.684 | C13804 | ORG 20599 | 1.071 | 0.746 | 0.012 |
|  | 385.344-7.886 | C13550 | Cerebrosterol | 2.976 | 8.236 | 0.046 |
|  | 359.153-8.322 | C13779 | XE991 | 2.587 | 3.282 | 0.028 |
|  | 361.150-8.322 | C01441 | Neamine | 3.163 | 4.633 | 0.023 |
|  | 305.214-8.519 | C05294 | 19-Hydroxytestosterone | 1.896 | 1.669 | 0.049 |
|  | 351.229-8.533 | C06429 | Docosahexaenoic acid(DHA) | 1.113 | 1.331 | 0.033 |
|  | 709.559-8.548 | C17569 | Ubiquinone-8 | 7.038 | 0.019 | 0.002 |
|  | 343.195-8.563 | C00219 | Arachidonic acid | 2.132 | 1.757 | 0.015 |
|  | 343.149-8.577 | C13847 | CP 339818 | 1.599 | 1.464 | 0.027 |
|  | 499.301-8.577 | C08806 | Cucurbitacin S | 1.939 | 1.627 | 0.016 |
|  | 604.404-8.592 | C20584 | Lolicine A | 1.488 | 1.377 | 0.041 |
|  | 623.366-8.592 | C11606 | NAc-FnorLRF-amide | 1.744 | 1.513 | 0.014 |
|  | 363.287-8.628 | C19427 | Glycidyltearate | 1.661 | 2.338 | 0.023 |
|  | 351.288-8.705 | C02982 | L-2-Hydroxyphytanate | 1.712 | 2.317 | 0.028 |
|  | 363.236-8.720 | C14630 | 9alpha-Fluoro-11beta-hydroxy-6alpha-methylpregn-4-ene-3,20-dione | 2.112 | 2.020 | 0.014 |
|  | 389.302-8.795 | C15422 | 2alpha-Methyl-17beta-[(tetrahydro-2H-pyran-2-yl) oxy]-5alpha-androstan-3-one | 1.517 | 1.846 | 0.046 |
|  | 339.288-8.839 | C13859 | 1-O-Hexadecyl-sn-glycerol | 1.051 | 1.846 | 0.021 |
|  | 410.790-8.839 | C18109 | 1,2,3,6,7,8-Hexachlorodibenzofuran | 3.135 | 4.289 | 0.028 |
|  | 391.284-8.943 | C01558 | Bile acid | 1.342 | 1.470 | 0.037 |
|  | 365.194-8.994 | C09155 | Phorbol | 1.464 | 1.483 | 0.029 |
|  | 379.319-9.099 | C19623 | 22-Hydroxydocosanoate | 1.447 | 1.344 | 0.028 |
|  | 365.303-9.159 | C13860 | 1-O-Octadec-9-enyl glycerol | 1.118 | 1.652 | 0.037 |
|  | 655.574-9.204 | C18138 | 2,2',4,4',5,6'-Hexabromodiphenyl ether | 1.377 | 1.382 | 0.028 |
|  | 419.350-9.386 | C06341 | 7alpha,27-Dihydroxycholesterol | 1.509 | 2.112 | 0.032 |
|  | 425.339-9.728 | C03594 | 7alpha-Hydroxycholesterol | 2.029 | 2.228 | 0.006 |
|  | 519.331-9.728 | C08803 | Cucurbitacin O | 1.084 | 1.265 | 0.016 |

| ESI (+)/ESI (-) | 927.675-8.577 | C15956 | Thermozeaxanthin-13 | 1.649 | 1.508 | 0.023 |
| --- | --- | --- | --- | --- | --- | --- |
|  | 353.303-9.039 | C19625 | Docosanedioate | 1.382 | 2.054 | 0.039 |
| ESI (-) | 269.210-10.292 | C19614 | 16-Oxo-palmitate | 1.993 | 0.586 | 0.038 |
|  | 295.227-10.292 | C14767 | (9S)-Hydroxyoctadecadienoic acid | 1.707 | 0.702 | 0.020 |
|  | 331.190-7.028 | C11857 | Gibberellin A12 | 1.138 | 1.619 | 0.045 |
|  | 483.311-7.363 | C08971 | Propapyriogenin A2 | 1.614 | 2.999 | 0.045 |
|  | 317.210-7.655 | C00909 | Leukotriene A4 | 1.364 | 1.623 | 0.020 |
|  | 339.199-7.655 | C15090 | 17-Hydroxy-3-oxo-17alpha-pregna-1,4-diene-21-carboxylic acid, gamma-lactone | 1.355 | 1.241 | 0.018 |
|  | 522.260-7.841 | C11309 | Calcimycin | 1.340 | 1.384 | 0.030 |
|  | 582.327-7.691 | C20731 | Penitrem B | 1.792 | 1.695 | 0.011 |
|  | 593.354-8.018 | C16147 | Glycosyl-4,4'-diaponeurosporenoate | 2.574 | 3.512 | 0.005 |
|  | 1091.612-8.032 | C16885 | Gambieric acid A | 1.529 | 1.803 | 0.010 |
|  | 333.245-8.176 | C13713 | 3alpha,12alpha-Dihydroxy-5beta-pregnan-20-one | 1.317 | 1.461 | 0.022 |
|  | 384.254-8.204 | C08703 | Ovatine | 2.430 | 2.109 | 0.005 |
|  | 315.195-8.218 | C14717 | 15-Deoxy-delta-12,14-PGJ2 | 1.834 | 1.942 | 0.005 |
|  | 404.280-8.253 | C08670 | Cassaine | 4.395 | 4.165 | 0.004 |
|  | 528.274-8.282 | C10831 | Zygadenine | 1.135 | 1.704 | 0.031 |
|  | 618.342-8.311 | C20548 | Lolitriol | 2.595 | 5.069 | 0.033 |
|  | 319.189-8.325 | C00390 | Ubiquinol | 2.203 | 2.424 | 0.038 |
|  | 431.173-8.339 | C11784 | Echitovenine | 2.113 | 2.047 | 0.030 |
|  | 449.147-8.339 | C09478 | Auriculoside | 2.563 | 2.500 | 0.027 |
|  | 517.138-8.339 | C16224 | Medicarpin 3-O-glucoside-6'-malonate | 2.824 | 2.853 | 0.010 |
|  | 301.215-8.353 | C12083 | (5Z,7E,9E,14Z,17Z)-Eicosapentaenoate | 3.001 | 3.042 | 0.017 |
|  | 393.203-8.353 | C09760 | Kazinol A | 2.833 | 2.317 | 0.005 |
|  | 289.216-8.328 | C04295 | Androstenediol | 2.718 | 3.058 | 0.048 |
|  | 434.273-8.439 | C20587 | Paspaline B | 3.590 | 4.101 | 0.030 |
|  | 550.264-8.439 | C05122 | Taurocholate | 1.654 | 1.936 | 0.023 |
|  | 241.215-8.511 | C16665 | 12-Methyltetradecanoic acid | 1.102 | 0.769 | 0.035 |
|  | 371.179-8.511 | C05552 | Biocytin | 1.024 | 0.806 | 0.030 |
|  | 463.207-8.525 | C02808 | Acetylblasticidin S | 1.992 | 0.610 | 0.024 |
|  | 475.163-8.525 | C12276 | Calcium pantothenate | 1.362 | 0.732 | 0.018 |
|  | 531.196-8.525 | C08760 | Glaucarubin | 1.019 | 0.802 | 0.026 |
|  | 543.151-8.525 | C12384 | Premithramycin A1 | 2.026 | 0.598 | 0.030 |
|  | 395.219-8.539 | C15989 | (9S,10S)-10-Hydroxy-9-(phosphonooxy)octadecanoate | 1.361 | 0.759 | 0.022 |
|  | 446.133-8.539 | C15665 | Ro 18-5364 | 1.878 | 1.493 | 0.008 |
|  | 457.187-8.539 | C17614 | 1-Octen-3-ol-3-o-beta-D-xylopyranosyl (1->6)-beta-D-glucopyranoside | 1.506 | 0.714 | 0.030 |
|  | 757.401-8.539 | C08919 | Yamogenin 3-O-neohesperidoside | 1.744 | 0.662 | 0.021 |

|  | 425.163-8.553 | C09261 | Disenecionyl cis-khellactone | 1.154 | 1.299 | 0.007 |
| --- | --- | --- | --- | --- | --- | --- |
|  | 427.158-8.553 | C10458 | Furcatin | 1.557 | 1.441 | 0.009 |
|  | 416.316-8.571 | C12272 | N-Oleoyl dopamine | 2.167 | 3.359 | 0.024 |
|  | 440.316-8.568 | C19805 | AP1 | 1.246 | 1.865 | 0.029 |
|  | 973.661-8.571 | C15955 | Thermocryptoxanthin-15 | 2.078 | 1.468 | 0.006 |
|  | 259.241-8.588 | C01554 | 5alpha-Androstane | 1.634 | 1.379 | 0.030 |
|  | 273.211-8.588 | C02940 | 3-Oxo-5alpha-steroid | 2.114 | 1.529 | 0.008 |
|  | 306.241-8.588 | C20033 | Pumiliotoxin A | 1.430 | 1.311 | 0.023 |
|  | 403.156-8.588 | C10511 | Osajin | 2.900 | 2.096 | 0.005 |
|  | 453.161-8.588 | C01937 | Methotrexate | 1.686 | 0.701 | 0.008 |
|  | 569.166-8.588 | C18004 | 5''-Phosphoribostamycin | 1.823 | 0.538 | 0.045 |
|  | 709.401-8.588 | C11295 | NAc-L4Y-amide | 1.162 | 0.778 | 0.023 |
|  | 303.232-8.603 | C15176 | 17-Methyl-5alpha-androst-2-ene-lalpha,17beta-diol | 1.537 | 1.342 | 0.030 |
|  | 827.402-8.588 | C08952 | Gypsogenin 3-O-rhamnosylglucuronide | 3.621 | 0.288 | 0.022 |
|  | 439.208-8.603 | C14645 | Estradiol-17-phenylpropionate | 1.026 | 0.817 | 0.037 |
|  | 379.156-8.617 | C19520 | Scarlet Red | 1.600 | 1.587 | 0.048 |
|  | 398.132-8.617 | C11234 | Difloxacin | 1.512 | 1.475 | 0.045 |
|  | 605.455-8.617 | C15895 | 3,4-Dihydrospheroidene | 1.086 | 1.204 | 0.030 |
|  | 518.286-8.646 | C20546 | Terpendole C | 1.904 | 1.916 | 0.035 |
|  | 362.236-8.746 | C08667 | Cardiopetalidine | 1.482 | 0.567 | 0.005 |
|  | 305.247-8.774 | C03242 | (8Z,11Z,14Z)-Icosatrienoic acid | 1.630 | 1.519 | 0.031 |
|  | 355.157-8.817 | C09308 | Rutamarin | 1.531 | 1.569 | 0.044 |
|  | 391.208-8.817 | C06497 | WIN I(S) | 1.257 | 1.277 | 0.038 |
|  | 293.248-8.831 | C08366 | Sterculic acid | 1.537 | 1.476 | 0.046 |
|  | 355.262-8.846 | C16185 | (4Z,7Z,10Z,13Z,16Z,19Z)-Docosahexaenoic acid ethyl ester | 1.659 | 1.724 | 0.016 |
|  | 428.317-8.874 | C10808 | Imperialine | 1.792 | 1.812 | 0.018 |
|  | 307.262-9.009 | C16525 | Icosadienoic acid | 1.767 | 1.819 | 0.006 |
|  | 375.250-9.009 | C15157 | 3-Acetyl-5alpha-androstane-3beta,17beta-diol 3-acetate | 1.797 | 1.852 | 0.006 |
|  | 505.210-9.009 | C01593 | Limonoate | 1.317 | 1.494 | 0.034 |
|  | 269.246-9.024 | C16995 | Methyl palmitate | 1.413 | 1.468 | 0.018 |
|  | 337.326-9.024 | C14775 | 14,15-DHET | 1.702 | 1.557 | 0.029 |
|  | 399.206-9.024 | C17648 | Cinncassiol B | 1.352 | 1.375 | 0.018 |
|  | 445.212-9.080 | C04036 | 1-Palmitoylglycerol 3-phosphate | 1.175 | 1.227 | 0.013 |
|  | 430.332-9.181 | C10830 | Peimine | 1.931 | 2.218 | 0.033 |
|  | 805.436-9.209 | C08947 | Gymnemic acid I | 1.420 | 0.734 | 0.041 |
|  | 381.195-9.224 | C17645 | Cinncassiol A | 2.222 | 1.932 | 0.008 |
|  | 399.184-9.224 | C14668 | Cortancyl | 1.713 | 1.499 | 0.007 |
|  | 283.262-9.238 | C01530 | Octadecanoic acid | 1.134 | 1.207 | 0.007 |
|  | 297.278-9.416 | C16535 | Nonadecanoic acid | 1.828 | 1.684 | 0.027 |
|  | 427.238-9.416 | C10011 | Nummularine F | 1.597 | 1.603 | 0.032 |

*Abbreviations:* m/z – RT, MS and retention time; VIP, variable importance for projection; F.C., fold change; q-value, adjusted p value calculated by two-tailed Wilcoxon rank-sum tests after false discovery rate correction.
